# Methanol extract and fraction of *Anchomanes difformis *root tuber modulate liver mitochondrial membrane permeability transition pore opening in rats

**Published:** 2020

**Authors:** Oludele John Olanlokun, Kemi Oloke, Olufunso Olabode Olorunsogo

**Affiliations:** 1 *Laboratories for Biomembrane Research and Biotechnology, Department of Biochemistry, Faculty of Basic Medical Sciences, College of Medicine, University of Ibadan, Nigeria*

**Keywords:** Anchomanes difformis, Phytoconstituents Mitochondrial ATPase, Lipid peroxidation, Cytochrome c, Mitochondrial permeability Transition pore opening

## Abstract

**Objective:**

Extracts of *Anchomanes difformis* (AD) are used in folkloric medicine to treat several diseases and infections. However, their roles in mitochondrial permeability transition pore opening are not known.

**Materials and Methods:**

The viability of mitochondria isolated from Wistar rat liver used in this experiment, was assessed by monitoring their swelling amplitude in the absence of calcium and reversal of calcium-induced pore opening by spermine. The effects of methanol extract and fraction of *A. difformis *(MEAD and MFAD, respectively) on Mitochondrial Membrane Permeability Transition (MMPT) pore opening, ATPase activity, cytochrome c release and ferrous-induced lipid peroxidation were assessed spectrophotometrically. Phytochemical constituents of MEAD and MFAD were assessed using Gas Chromatography- Mass Spectrometry (GC-MS).

**Results:**

The MEAD (10, 20, 40 and 80 μg/ ml) had no effect on MMPT pore opening in the absence of Ca^2+^, whereas MFAD at 80 μg/ml had a large amplitude pore opening effect. Both MEAD and MFAD reversed Ca^2+^‌‌-induced swelling with inhibition values of 18, 21, 24, 23% (for MEAD) and 41, 36, 35, and 26% (for MFAD) at 10, 20, 40 and 80 μg/ml, respectively. MFAD significantly enhanced F_1_F_0_ ATPase activity and caused cytochrome c release. Both MEAD and MFAD significantly inhibited ferrous-induced lipid peroxidation by 33.0, 64.0, 66, and 75% (for MEAD) and 24, 25, 30, and 45% (for MFAD), respectively. The GC-MS results revealed the presence of squalene as one of the major constituents of MEAD.

**Conclusion:**

These findings suggest that MFAD can be used to induce cell death via mitochondrial permeability transition in isolated rat liver. Inhibition of lipid peroxidation by MEAD and MFAD showed that the pore opening effect of the extract and fraction was not mediated via peroxidation of mitochondrial membrane lipids.

## Introduction

Traditional medicine commonly uses herbs for treatment of different diseases, all over the world. Plants were proven to be good sources of natural products which have been described as the active ingredients responsible for their therapeutic potentials. A good example of these plants is *Anchomanes difformis *(AD). It is a plant of the family Araceae that is commonly found in Africa especially in the sub Saharan African countries such as Nigeria, Ghana, Togo, Ivory Coast and Senegal (Ataman and Idu, 2015 ▶). Different preparations of the root tuber of this plant are believed to cure malaria, dieresis, diabetes and tuberculosis. It is also used against oral and anal lesions (Bero et al., 2009 ▶). The mechanisms of action of some medicinal plants in treating some disease conditions include but not limited to: perturbation of the membranes of microbial organisms (Sanchez et al., 2010 ▶), modulation of protein synthesis (Adnan et al., 2017 ▶) and induction of mitochondrial dysfunction in cancer cell-selective death (Wiench et al., 2012 ▶). Lately, mitochondria have become a target for the discovery of drugs that can enhance cell death. This is because the release of some mitochondrial proteins makes the death of cells inevitable (Ankur et al., 2012 ▶).

Mitochondria can show increase in their permeability to molecules of higher masses as a result of loss of transmembrane potential (ψm), increased mitochondrial matrix swelling, and rupture of the outer mitochondrial membrane (Baines et al., 2007 ▶). This process is called the mitochondrial membrane permeability transition (MMPT). Exposure of mitochondria to high levels of exogenous Ca^2+^, reactive oxygen species and high levels of inorganic phosphate are capable of causing the permeabilisation of the mitochondria (Seidlmayer et al., 2015 ▶). During mitochondrial permeabilisation, the proton-motive force is disrupted as a result of the opening of the pore. Although, there had been series of models about the components of the pore, recent evidence has shown that the c ring of F_1_F_0_ ATPAse is plausibly one of the components of the pore (Bonora and Pinton, 2014 ▶). Therefore, any drug or drug candidate that modulates the enhancement of F_1_F_0_ ATPase, may likely modulate the pore.

Transient opening of the MMPT pore further results in depolarisation of the inner mitochondrial membrane, swelling of mitochondrial matrix, rupture of outer mitochondrial membrane (Kinnally and Antonsson, 2007 ▶), and release of pro-apoptotic proteins such as cytochrome c into the cytosol thus causing apoptosis. In addition to these effects, oxidative phosphorylation is uncoupled and inorganic phosphate concentration increases via the stimulation of ATP hydrolysis by F_1_F_0_ ATPase. The MMPT pore has been a target for drug development in diseases where there is deregulation of apoptosis. Experimental evidence indicates that certain bioactive agents present in medicinal plants such as quercetin in onions, capsaicin in chili pepper, etc. can modulate apoptosis through permeabilisation of the mitochondrial membrane (Martin, 2006 ▶). There is therefore increased interest globally for evaluation of potent bioactive agents that may interact with MMPT pore, in order to develop drugs that may upregulate apoptosis in the treatment of tumor, hyperplasia or cancer. 

*A. difformis *(Blume) Engl. is a herbaceous plant with thorny stem having huge divided leaves and spathe that arise from a horizontal tuber occurring in the forest of West Africa. The plant has rhizome and grows in tropical African forests and is mostly found in moist and shady places. In French Guinea, the tuber (rhizome) is used medicinally for topical application to increase blood circulation; a decoction of the tuber is used in cough treatment and ulcer while the peeled tuber, soaked in water, is used in treating cases of dysentery. The tuber has also been used as an antidiabetic, antituberculosis, and antimalarial agent, and against oral and anal lesions. In some part of Africa such as Tanzania, the juice from the root tuber is used as eye drop in the treatment of river blindness, or as a diuretic and purgative agent (Oghale and Idu, 2016 ▶). Also, in combination with *Cissus quadrangularis*, AD is used for treatment of prostate cancer in Cameroon (Noumi, 2010 ▶). In spite of different folkloric use of this plant, limited knowledge about its interaction with mitochondria necessitates a scientific research to either explore more of its medicinal use or present a scientific caution on its use.

## Materials and Methods

**Collection of plant material**

Whole tubers of *A. difformis* (AD) were obtained from the uncultivated area at Ado-Ekiti. The specimen was identified by Mr. F.O. Omotayo of Plant Herbarium, Ekiti State University, Ado-Ekiti and a voucher number (UHAE 2017/065) was obtained.

**Extraction of plant material**

The processing of the root tubers of AD and extraction was done as described earlier (Handa et al., 2008 ▶). Briefly, the root tubers of AD were washed, peeled, sliced into pieces and air-dried at room temperature and pulverised using a blender. The powdered root tuber was soaked in methanol for 3 days: the crude methanol extract was concentrated under reduced pressure at 40^o^C and the concentrated residue was heated in a water bath at 50^o^C to obtain a solvent-free extract. The methanol extract was used to obtain methanol fraction of the extract using vacuum liquid chromatography (VLC). The resultant was concentrated using a rotary evaporator under reduced pressure at 40ºC, and the residues were transferred to separate bottles and stored in a refrigerator until use. 

**Experimental animals**

Male albino rats (90±4 g) were obtained from the Preclinical Animal House, College of Medicine, University of Ibadan, Nigeria, and kept at the Biochemistry Department Animal house, University of Ibadan, under light-controlled conditions (12 hr-light/12 hr dark cycle) and in well-ventilated plastic cages. The animals were given water and rat chow *ad-libitum.*

**Ethical consideration in animal handling**

All experiments involving the use of animals in this article, were performed in accordance with the public health policy on Human Care and Use of Laboratory Animals of National Institute of Health (NIH, 1985 ▶).

**Preparation of low-ionic-strength liver mitochondria**

Low-ionic-strength liver mitochondria were isolated from male albino rats using the method described by Johnson and Lardy (1967) ▶. The animals were sacrificed by cervical dislocation, dissected and the livers were excised, washed several times in isolation buffer (210 mM Mannitol, 70 mM Sucrose, 5 mM Hepes-KOH (pH 7.4) and 1 mM EGTA), weighed and minced using a pair of scissors. A 10% suspension was prepared by homogenising the liver on ice using a Teflon-glass cup homogeniser in isolation buffer. The liver homogenate was loaded into a refrigerated Sigma 3-30 K centrifuge, where the nuclear fraction and cell debris were sedimented at low speed centrifugation twice at 2,300 rpm for 5 min each time. The supernatant was further centrifuged at 13,000 rpm (10,000 g) for 10 min to pellet the mitochondria. The mitochondrial pellets obtained were washed in washing buffer (210 mM mannitol, 70 mM sucrose, 5 mM Hepes-KOH (pH 7.4) and 0.5% bovine serum albumin (BSA)) and centrifuged twice at 12,000 rpm for 10 min each time. The mitochondria were immediately suspended in suspension buffer (210 mM mannitol, 70 mM sucrose, and 5 mM Hepes-KOH (pH 7.4) then dispensed in Eppendorf tubes in aliquot and kept at 4ºC. Mitochondria used for the determination of ATPase activity and lipid peroxidation, were isolated as described above except that 0.25 M sucrose was used for the isolation of the mitochondria.

**Protein determination**

The mitochondrial protein was estimated according to the method of Lowry et al. (1951) ▶ using Bovine Serum Albumin (BSA) as standard.

**Assessment of mitochondrial membrane permeability transition in rat liver mitochondria**

Changes in the volume of isolated liver mitochondria were measured quantitatively at 540 nm based on the procedure described by Lapidus and Sokolove (1993) ▶. Mitochondria (0.4 mg of protein/ml) were pre-incubated in a 1 cm light path glass cuvette in the presence of 0.8 μM rotenone in suspension buffer for 3 min at 30°C prior to the addition of succinate. After 30 sec of this incubation, 5 mM sodium succinate was added to energise the reaction and mitochondrial permeability was quantified as changes in absorbance at 540 nm over a period of 12 min at 30 sec interval. To assess the pore opening effects of calcium, mitochondrial were pre-incubated in rotenone and suspension buffer for 3 min after which 3 µM CaCl_2_ was added. Sodium succinate was added 30 sec later. To assess the reversal effect of spermine, mitochondria were pre-incubated in rotenone, suspension buffer and 4 mM spermine for 3 min after which 3µM Cacl_2 _and 5 mM sodium succinate were added as previously described. To assess the pore opening effects of MEAD and MFAD, varying concentrations of the extract and fraction were added to the assay medium and the change in absorbance was monitored for 12 min with 30 sec intervals, at 540 nm using 752 N UV visible spectrophotometer.

**Determination of mitochondrial ATPase activity**

Mitochondrial ATPase activity was determined using the method of Lardy and Wellman (1953) ▶. Each test tube (in triplicate) contained 65 mM Tris-HCl buffer (pH 7.4), 0.5 mM KCl and 25 mM sucrose in a reaction volume of 1 ml. Varying concentrations of MEAD and MFAD were added to the designated tubes and the solutions were made up to 2000 µl with distilled water. The ATP (1 mM) was added to the designated tubes and the whole set up was transferred to a shaking water bath at 27ºC. Mitochondria were added to the zero time and the reaction was stopped immediately by the addition of 1 ml of 10% sodium dodecyl sulphate (SDS). Mitochondria were added to the rest of the test tubes except the blank and ATP-only every 30 sec. The 2, 4-dinitrophenol (25 µM DNP) was added to the uncoupler tube and just immediately mitochondria were added. The reaction was stopped by addition of 1 ml (10%) SDS to each test tube (except zero time) every 30 sec. After 30 min of incubation in the water bath at 27^o^C, 1 ml of the reaction mixture was taken for phosphate determination. Distilled water (4 ml) was added to the 1 ml in a test tube to dilute it. This was followed by the addition of 1 ml of 1.25% ammonium molybdate and 1 ml of a 9% freshly prepared solution of ascorbate. The tubes were thoroughly mixed, gently shaken and allowed to stand for 30 min. A standard solution of 1mM potassium dihydrogen phosphate was similarly treated for phosphate standard curve. The intensity of the blue colour was read at 660 nm using a 752 N UV visible spectrophotometer. The standard phosphate curve was plotted and the concentration of inorganic phosphate released per milligram protein per minute, was calculated.

**Assessment of cytochrome c release**

Cytochrome c release was assessed as described by Appaix et al. (2000) ▶. Isolated mitochondria were incubated with suspension buffer, 0.8 µM rotenone, various concentrations of MEAD and MFAD and 5 mM succinate while the control test tubes were treated with 12 mM CaCl_2._ The extract and fraction control did not contain mitochondria. The mixtures were incubated for 30 min at 25^o^C after which, they were centrifuged at 13,000 rpm. The absorbance of the supernatant was read at 414 nm. The absorbance of extract and fraction control was deducted from that of the test groups. Cytochrome c was used as standard.

**Determination of lipid peroxidation**

A modified thiobarbituric acid reactive species (TBARS) assay described by Ruberto et al., (2000) ▶ was used to measure the extent of lipid peroxidation using mitochondria as lipid-rich media, Briefly, a specific volume of mitochondria, equivalent to 1 mg/ml protein, was added to graded concentrations of the MEAD and MFAD in the test tube and the volume was then made up to 1 ml with distilled water. Thereafter, 0.05 ml of 60 µM FeSO_4_ was added to induce lipid peroxidation and the mixture was incubated at 37^o^C for 30 min. Then, 1.5 ml of acetic acid, followed by 1.5 ml of TBA in SDS (0.8 in 1.1% respectively) was added. The resulting mixture was vortexed and heated at 95^o^C for 60 min. After cooling, 5 ml of butan-1-ol was added to each tube and centrifuged at 3,000 rpm for 10 min. The absorbance of the organic upper layer was read at 532 nm and the percentage of inhibition of lipid peroxidation by MEAD and MFAD was calculated using the following formula:

Percentage inhibition of lipid peroxidation = (Ab.Control – Ab. Test/ Ab. Control) x 100

Ab stands for absorbance.

**Identification of essential components of plant extract using gas chromatography mass spectrometry (GC-MS).**

Gas chromatography-mass spectrometry (GC-MS) analysis of the ME and MF was carried out using an Agilent 7890n gas chromatograph hyphenated with an Agilent mass detector triple Quad 7000A in EI mode at 70Ev (m/z range 40-600 amu) with an ion source temperature of 250^o^C and an Agilent ChemStation data system. The GC column was equipped with an HP-5MS column (30 m×250 µm×0.25 µm) a split-splitless injector heated at 200^o^C and a Flame Ionization Detector (FID) at 230^o^C. Oven temperature was programmed as follows: Initial temperature 40^o^C for 5 min, increased 5^o^C/min to 180^o^C for 6 min and then 10^o^C/min to 280^o^C for 12 min. Carrier gas was helium at a flow rate of 1 ml/min. Injection volume was 2 µl (split ratio 1:20)

The GC-MS QP 2010 Plus was used for the analyses of plants with Ion source and interface temperature at 250^o^C; solvent cut time 2.5 min with relative detector gain mode and threshold 3000; scan MS ACQ mode; detector FTD; mass range of m/z 40-400.

Identification of the essential components was done based on their retention indices along with comparison of their mass spectral fragmentation patterns by computer matching with in-built data and commercial libraries. Other search libraries used included database/NIST08. L. 

**Statistical analysis**

Representative profile of four similar determinations were used for the mitochondrial permeability transition assays. For other assays, data were expressed as mean±SD of triplicate readings. Values were analysed using one-way ANOVA followed by Tukey’s *post hoc* comparison among data in columns using GraphPad prism 6.0 and a p˂0.05 was considered to be statistically significant.

## Results

**Effects of MEAD on MMPT in the absence and presence of calcium**

**Figure 1:**
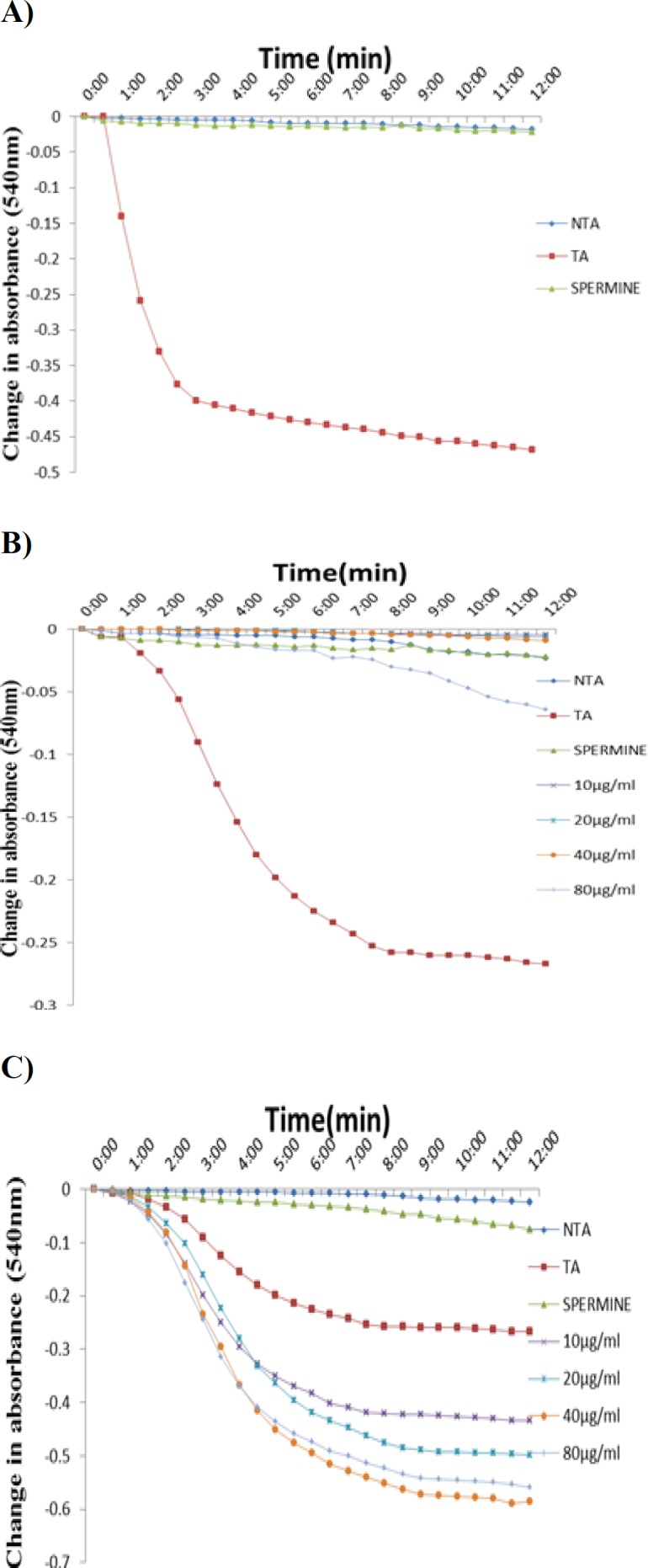
Representative profile for the assessment of isolated rat liver mitochondrial permeability transition pore opening. Figure 1A shows the assessment of the mitochondria integrity in the absence of calcium, in the presence of calcium and reversal of calcium-induced mitochondrial membrane permeability transition pore opening by spermine. Figures 1B and 1C show the effect of varying concentrations of MEAD in the absence (B) and in the presence (C) of calcium on the mitochondrial membrane permeability transition pore opening. NTA: no triggering agent; TA: triggering agent

Figures 1 A, B and C show the integrity of the isolated mitochondria, the effects of the methanol extract (MEAD) on MMPT in the absence of calcium (1B) and in the presence of calcium (1C), respectively. Figure 1A shows that there were no significant changes in the volumes of intact mitochondria respiring on succinate in the absence of calcium as shown by little changes in light scattering effect of the mitochondria at 540 nm. Upon the addition of calcium, there was an induction of opening of the mitochondrial membrane permeability transition pore. Spermine, a standard inhibitor of calcium-induced MMPT pore opening, reversed the opening of the pore. This result shows that mitochondria were intact in the absence of calcium but exogenous calcium induced MMPT pore, while spermine significantly reversed the Ca^2+^-induced opening of the pore of mitochondria respiring on succinate. This indicated that the membrane integrity of the liver mitochondria was intact, not uncoupled and hence, suitable for further use. In this context, Figure 1A shows the suitability of the isolated mitochondria for the mitochondria permeability transition pore opening assay. The results obtained revealed that MEAD has no significant effect on the opening of MMPT pore at all concentrations used, in the absence of calcium (Figure 1B). This extract however, in the presence of calcium, potentiated calcium-induced pore opening (Figure 1C). 

**Effects of MFAD on MMPT in the absence and presence of calcium**

**Figure 2:**
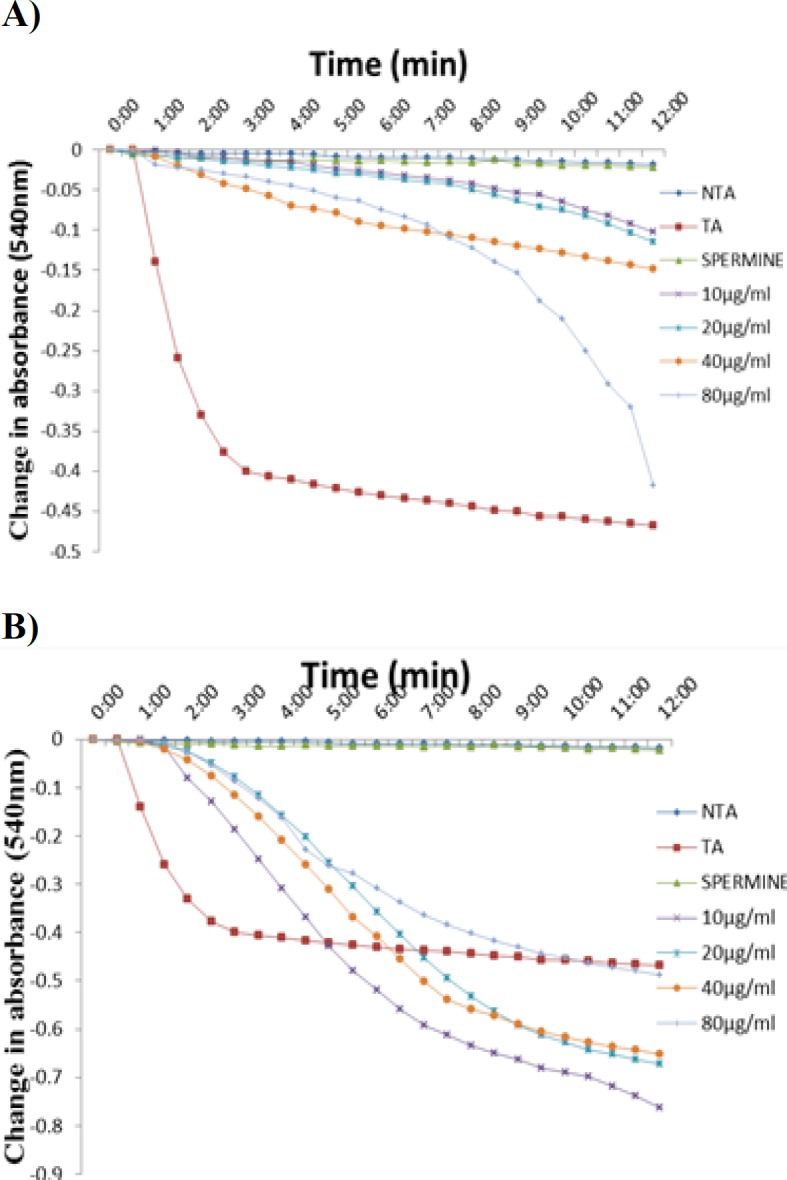
Representative profile showing the effects of MFAD on the mitochondrial permeability transition pore opening in the absence (A) and in the presence (B) of calcium. NTA: No triggering agent, TA: Triggering agent

Figures 2 A and B show the effects of MFAD on MMPT in the absence (2A) and presence (2B) of calcium. The results obtained showed that MFAD was able to induce pore opening at the highest concentration used (80 μg/ml) in the absence of calcium. MFAD however had a reversal effect on calcium-induced pore opening as the concentration increased.

**Effects of MEAD and MFAD on mitochondrial ATPase activity and cytochrome c release**

**Figure 3:**
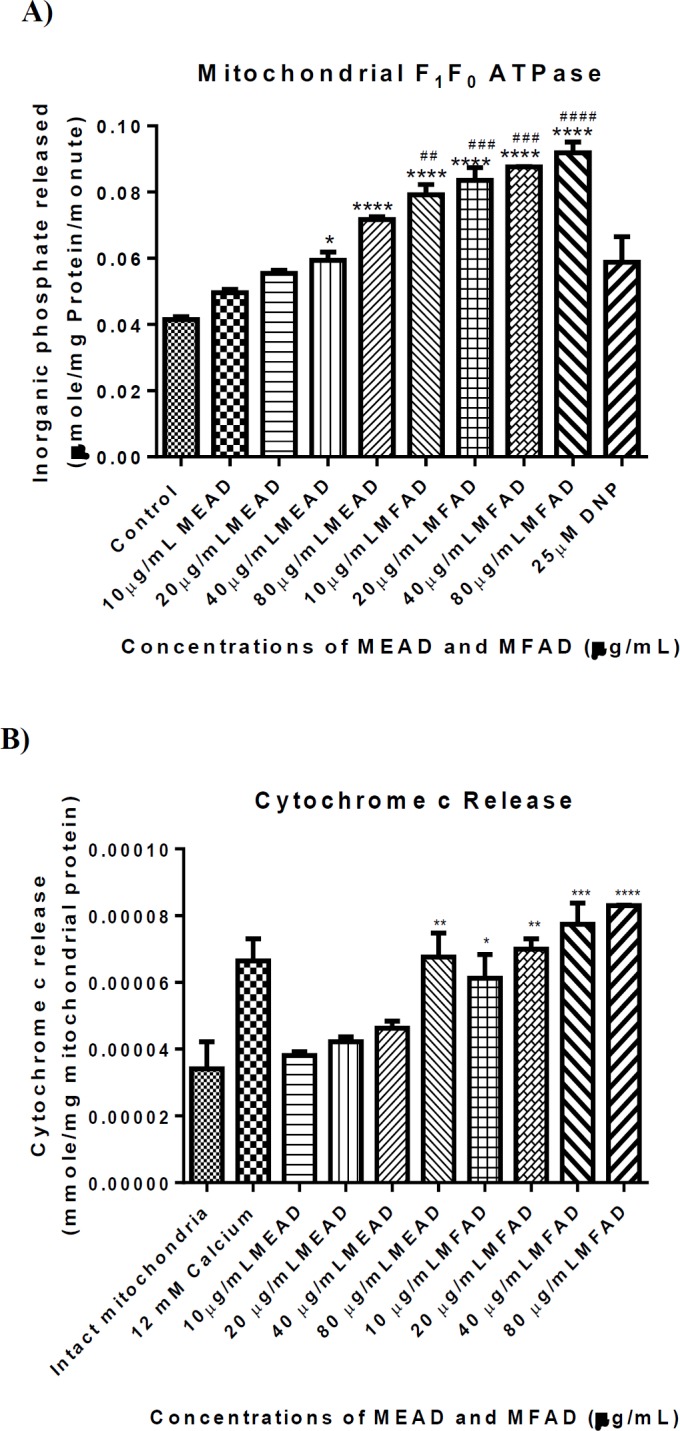
Effect of MEAD and MFAD on F_1_F_0_ ATPase activity (Figure 3A) and cytochrome c release (Figure 3B) from rat liver mitochondria at physiological pH (7.4) (*^, #^ p<0.05, **^,^^ ##^ p<0.01, ***^,^^###^ p<0.001 all vs control (*) and DNP (^#^)). In Figure 3B, there is a significant increase in the level of cytochrome c release at all concentrations of MFAD used when compared with the intact mitochondria. Bar represented the mean±SD. (n=4). (*p<0.05, ** p<0.01, *** p<0.001 all vs intact mitochondria)

The F_1_F_0_ ATPase activity was monitored in the presence of both MEAD and MFAD. The results obtained showed that MEAD enhanced the ATPase activity relative to the control. Also, MFAD enhanced ATPase activity at the physiological pH (Figure 3A) in a concentration-dependent manner with the maximum enhancement at 80 µg/ml. The levels of cytochrome c release induced by both MEAD and MFAD are shown in Figure 3B. Based on this Figure, there was a significant (p˂0.001) increase in cytochrome c release induced by MFAD relative to intact mitochondria while only the highest concentration of MEAD significantly (p˂0.01) induced an increase in cytochrome c release.

**Effects of MEAD and MFAD on lipid peroxidation**

**Figure 4:**
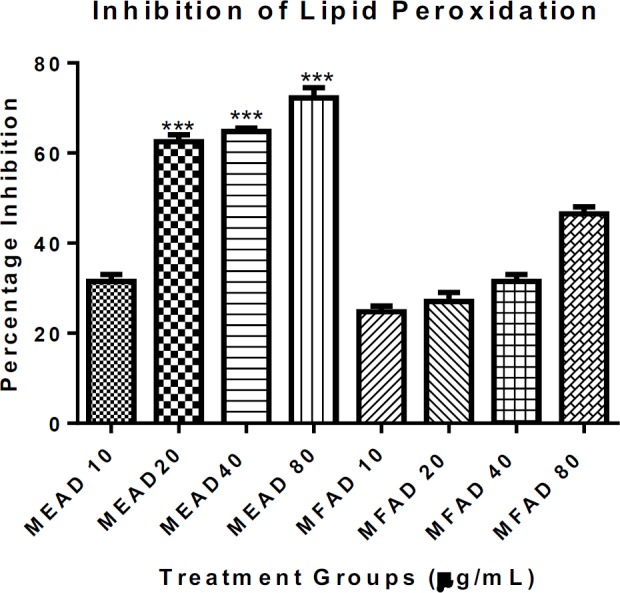
Effect of varying concentrations of MEAD and MFAD on Fe^2+^-induced lipid peroxidation in normal rat liver mitochondria. Inhibition of lipid peroxidation by MEAD were statistically significant when 20, 40 and 80 µg/ml of MEAD were compared with corresponding concentrations of MFAD. Bar represented the mean±SD. (n=4). (***p*<*0.001)

The percentage of inhibition of lipid peroxidation by MEAD and MFAD is shown in Figure 4. The results showed that, there was a significant increase in percentage of inhibition of Fe^2+^-induced lipid peroxidation at 10, 20, 40, and 80 µg/ml concentrations of both MEAD and MFAD with percentage inhibition of 33, 64, 65.5, 74.5% (for MEAD) and 23.5, 25, 30, and 45% (for MFAD) at 10, 20, 40 and 80 μg/ml, respectively. The results also showed that both MEAD and MFAD inhibited lipid peroxidation maximally at 80 µg/ml while MEAD inhibited the lipid peroxidation more than MFAD.

**The GC-MS of MFAD and MEAD**

Table 1 shows the phytochemicals present both in MEAD and MFAD. There are some phytochemicals present in MEAD that were not found in MFAD. This could be as a result of chromatographic partitioning of the methanol extract with various solvents before MFAD was obtained.

**Table 1a:** Essential composition of methanol extract of *Anchomanis difformis *root tuber

**S/NO **	**Compound**	**Abundance (%)**
**1**	Hexadecane	7.24
**2**	Heptadecane	7.32
**3**	Octadecane	6.44
**4**	Dodecane,2,6,10-trimethyl	4.17
**5**	Nonadecane	7.87
**6**	Hexadecanoic acid, methyl ester	7.94
**7**	Eicosane	7.60
**8**	9,12-octadecadienoic acid, methyl ester	23.41
**9**	Methyl stearate	4.79
**10**	Docosane	7.25
**11**	Tricosane	4.32
**12**	Squalene	11.64

**Table 1b:** Essential composition of methanol fraction of *Anchomanis difformis *root tuber

**S/N **	**Compound**	**Abundance (%)**
**1**	P-Dioxane-2-3-diol	7.76
**2**	Tetracontane,3,5,24-trimethyl	1.58
**3**	Pentadecane	3.53
**4**	Hexadecane	4.67
**5**	Tridecane	4.85
**6**	Heptadecane	5.30
**7**	Dodecane,2,6,11-trimethyl	3.39
**8**	Octadecane	5.02
**9**	Dodecane,2,6,10-trimethyl	2.51
**10**	Nonadecane	5.31
**11**	Hexadecanoic acid methyl ester	2.68
**12**	n-hexadecanoic acid	5.07
**13**	Eicosane	7.37
**14**	Heneicosane	13.25
**15**	Methyl 8,10-dimethyl hexadecanoate	3.34
**16**	9,10-octadecadienoic acid	7.80
**17**	Octadecane,1-ethenyloxy	3.65
**18**	Docasane	6.57
**19**	Tricosane	3.43
**20**	Tetracosane	2.92

## Discussion

Pharmacological approaches acting directly on the mitochondrial membrane by targeting mitochondrial channels permeabilisation or by stimulation of survival pathways such as the release of antiapoptotic proteins are good measures for the management of diseases where dysregulation of apoptosis had been implicated. 

Since its discovery, MMPT pore has been proposed to be an important regulator of cell death. This is because pharmacological modulations of the components of the pore will provide scientific explanations for various pathologies such as neurodegenerative, and cardiac diseases and cancer. Mitochondrial dysfunction, in particular, the induction of the pore, has been implicated in the cascade of events involved in the induction of apoptosis. It leads to mitochondrial depolarisation, uncoupling of mitochondria, uncoupling of oxidative phosphorylation, and large amplitude swelling, which in turn can lead to ATP depletion and cell death (Kroemer and Reed, 2000 ▶). Although, some drugs have been implicated in the modulation of the biological activities of mitochondria with the view of treating some diseases, some medicinal plants, which contain drug candidates, have been demonstrated to modulate the functional features of such established drugs. From time immemorial, medicinal plants have been known to contain metabolites known as phytochemicals with therapeutic uses. Certain bioactive agents, e.g. betulinic acid, in medicinal plants have been shown to elicit their chemopreventive effects through induction of mitochondrial pore (Pal et al., 2011 ▶). It is on this basis that we investigated the *in vitro* effects of MEAD and MFAD on MMPT pore for the first time.

 Traditionally, the root tubers of AD are infused with either water or alcoholic drinks for the treatment of some diseases such as inflammation, edema, cough, ulcer, diabetes mellitus and dysentery (Alabi et al., 2018 ▶). We used the MEAD and MFAD in this research as a variation to see if the method of extraction will enhance the traditionally acclaimed potency of the plant and also to represent both polar and non-polar characteristics of the extract.

The results obtained from this work showed that MFAD has an inductive effect on the MMPT in the absence of calcium and a reversal effect on calcium-induced opening of the pore. This means that like calcium, MFAD has the independent effect to induce the opening of the pore possibly by interacting with the pore components at the binding site(s) of calcium. Interestingly, this same fraction inhibited calcium-induced opening of the pore. This may be a result of competition for calcium binding site thus rendering calcium unavailable for pore-opening induction. Moreover, the finding that MEAD inhibited the opening of the pore in the presence of calcium, may possibly show that it has the ability to compete for calcium binding site as demonstrated by MFAD. The inability of the MEAD to induce pore opening effects at the same concentrations of MFAD that showed the inductive effect, implied that the phytochemicals shielding the active compound has been removed in the course of purification. We therefore conclude that purification enhanced the activity of MFAD and that both MEAD and MFAD can modulate the opening of the pore. 

Also, in this study, effects of both MEAD and MFAD on the activity of mitochondrial F_1_F_0_-ATPase and cytochrome c release were reported. The results obtained indicated that MEAD and MFAD enhanced ATPase activity maximally at the highest concentration (80 μg/ml) compared with the control group. The mitochondrial F_1_F_0_ ATPase or ATP synthase is known to use the proton gradient generated during the transfer of electron along the respiration chain and couple it to oxidative phosphorylation of ADP and inorganic phosphate to produce ATP required for diverse biochemical and cellular functions (Nelson et al., 2005 ▶). The collapse in mitochondrial membrane potential could result in the hydrolysis of ATP by F_1_F_0_-ATPase to overcome this bioenergetics challenge. This turns F_1_F_0_-ATPase into a consumer rather than a producer of ATP in failing cells. The studied effects of both MEAD and MFAD on F_1_F_0_ ATPase has shown that MFAD makes inorganic phosphate, a known inducer of the pore opening available for pore opening effect by enhancing its activity. Specifically, when ATP is hydrolysed to ADP and inorganic phosphate (Pi), the inorganic phosphate made available through this process, is an established inducer of the opening of the pore, therefore, an increase in the cytosolic concentration of Pi may further potentiate the opening of the pore (Baev et al., 2017 ▶). 

It is believed that reactive oxygen species are generated by complexes I and III of the inner mitochondria (Harper and Breton, 2004 ▶), due to the release of electrons by substrates such as NADH and FADH into the electron transport chain. In the course of oxidative process of ATP generation, mitochondria consume a high percentage of oxygen which during normal oxidative phosphorylation is converted to the superoxide radical (Shigenaga et al., 1994 ▶; Evans et al., 2002 ▶; Carreras et al., 2004 ▶). The generation and accumulation of ROS have been implicated as one of the mechanism through which cell death via apoptosis, can take place. This is because ROS can cause the peroxidation of the mitochondrial membrane phospholipids thus leading to cell death. Both MEAD and MFAD were tested for their effects on Fe^2+^_-_ induced lipid peroxidation in order to ascertain the exact cause of mitochondrial permeability transition. Data obtained from this study showed that varying concentrations of MEAD and MFAD have inhibitory effects on Fe^2+^-induced lipid peroxidation in a concentration-dependent manner. This suggests that the plant may contain phytochemical(s) that can protect the integrity of the mitochondrial membrane against oxidative damage. Although both drug candidates may be responsible for cell death, it is evidently clear in this study that such mechanism may not be mediated through the peroxidation of membrane lipids.

It is worthy to note that mitochondrial permeability transition is primarily triggered by matrix calcium overload. In addition to this, chemical agents that promote the oxidised state of pyridine nucleotides and xenobiotics that cause adenine nucleotide depletion, increased inorganic phosphate concentration and mitochondrial depolarisation, can cause mitochondrial permeability transition. Although, both MEAD and MFAD inhibited iron-induced lipid peroxidation in this study, it is interesting to note that both MEAD and MFAD significantly (p˂0.001) induced mitochondrial ATPase. It is believed that high concentrations of inorganic phosphate (Pi) inhibit mitochondrial oxidative phosphorylation and that accumulation of Pi and Ca^2+^ synergistically increase the permeability of inner mitochondrial membrane (Kowaltowski et al., 1996 ▶). Nguyen and coworkers (2015) ▶ discovered that permeability transition pore opening triggered by calcium overload was accelerated by further exposure of mitochondria to Pi uptake.

Gas chromatography-mass spectrometry (GC-MS) showed that MEAD and MFAD contain phytochemicals which can be of biological and pharmacological importance. The results obtained from phytochemical and GC-MS screening in this study, showed that both MEAD and MFAD contain a number of phytochemicals such as hydrocarbons, fatty acids and terpenes. It is interesting to note that squalene which is a major constituent of the MEAD (11.64 percent abundance), has been acclaimed to have anticancer, antioxidant, drug carrier and detoxifier properties both in animal models and *in vitro* (Kim and Faith, 2012 ▶). Several medicinal plants have shown similar cell death effects via cytotoxic potentials on cells; examples are *Scutellaria luteo-coerulea *(Motaez et al., 2015 ▶); *Perovskia abrotanoides (*Geryani et al., 2016 ▶) and *Cuscuta campestris* (Moradzadeh et al., 2018 ▶) but via different mechanisms.

We therefore, conclude that MFAD, in the absence of calcium, is capable of causing mitochondrial dysfunction; an important property that can be explored to commute deleterious cells to death. The modulatory effects of these extract and fraction on mitochondrial dysfunction may find application in situations that require control of cell death via apoptosis. Therefore, further work should therefore involve structural elucidation of the active components present in AD and their biological effects in mitochondrial-mediated cell death.
